# Grape polyphenols supplementation for exercise-induced oxidative stress

**DOI:** 10.1186/s12970-020-00395-0

**Published:** 2021-01-07

**Authors:** Edurne Elejalde, Mari Carmen Villarán, Rosa María Alonso

**Affiliations:** 1 TECNALIA, Basque Research and Technology Alliance (BRTA), Parque Tecnológico de Álava c/ Leonardo Da Vinci, 11, 01510 Miñano (Álava), Spain; 2 Analytical Chemistry Department, Faculty of Science and Technology, University of the Basque Country (UPV/EHU), P.O. Box 644, 48080 Bilbao, Spain

**Keywords:** Grape, Polyphenols, Antioxidants, Supplementation, Sport, Exercise, Oxidative stress

## Abstract

Exercise induces free radicals’ overproduction and therefore, an enhancement of oxidative stress, defined as an imbalance between the production of reactive species and the intrinsic antioxidant defense. Redox activity of reactive species plays an important and a positive role on exercise adaptation, but these species at very high concentrations have detrimental effects. As a result, the use of antioxidant supplements for reducing oxidative stress can be an effective health strategy to maintain an optimal antioxidant status. In this sense, grapes are an important source of natural antioxidants due to their high content in polyphenols. They have shown antioxidant potential benefits for the reduction of intense exercise effect in athletes of different sport disciplines. Consequently, it is plausible to hypothesize that a strategic supplementation with grape based products may be a good approach to mitigate the exercise induced oxidative stress. The goal of this review is to present the state of the art of supplementation effects with grape beverages and grape extracts on the oxidative stress markers in athletes. The data of polyphenolic dosages, participant characteristics and exercise protocols are reported.

## Background

The World Health Organization defines physical activity as any bodily movement produced by skeletal muscles that requires energy expenditure. Regular physical activity has significant health benefits at all ages. Conversely, physical inactivity (insufficient physical activity) is one of the leading risk factors for noncommunicable diseases (NCD) and death worldwide [1].

The scientific evidence is strong regarding how a physically active lifestyle decreases oxidative stress (OS) [2]. This reduction may be one of the mechanisms responsible for an attenuated cellular aging [3], increased insulin sensitivity and lipid profile regulation [4], and reduced endothelial dysfunction [5]. In fact, oxidative stress status is generally found to be lower in athletes than in sedentary individuals.

Nevertheless, several studies have also suggested that acute and strenuous bouts of aerobic and anaerobic exercise induce the overproduction of free radicals and therefore, an enhancement of OS [6]. This effect varies according to the exercise mode, volume, intensity, training level, age, sex or nutritional status [6–9]. As a result, the use of supplements with antioxidant properties [10] for reducing the oxidative stress may be an effective health strategy.

In this sense, there is growing interest in the use of polyphenol-rich fruit and vegetables to mitigate exercise induced physiologic stress [11–13]. And grapes are an evident example of a fruit with a high content in polyphenols and with an evident nutritional value. Table 1 details the nutrients present in grapes.

**Table 1:** Essential nutrients in 100 g of grapes

Nutrient^a^	Amount	Unit
Water	80.54	g
Energy	69	kcal
Protein	0.72	g
Total lipid (fat)	0.16	g
Carbohydrate	18.1	g
Fiber, total dietary	0.9	g
Sugars, total	15.48	g
**Minerals**		
Calcium, Ca	10	mg
Iron, Fe	0.36	mg
Magnesium, Mg	7	mg
Phosphorus, P	20	mg
Potassium, K	191	mg
Sodium, Na	2	mg
Zinc, Zn	0.07	mg
Copper, Cu	0.127	mg
Selenium, Se	0.1	mg
**Vitamins**		
Vitamin A	3	μg
Thiamin, Vitamin B1	0.069	mg
Rivoflavin, Vitamin B2	0.07	mg
Niacin, Vitamin B3	0.188	mg
Pyridoxine, Vitamin B6	0.086	mg
Folate, Vitamin B9	2	μg
Cyano-cobalamin, Vitamin B12	0	μg
Vitamin C	3.2	mg
Vitamin E	0.19	mg
Vitamin K	14.6	μg
**Phytonutrients**		
Carotene, alpha	1	μg
Carotene, beta	39	μg
Lutein-zeaxanthin	72	μg
Polyphenols, total (black)^b^	184.97	mg
Polyphenols, total (green)^c^	121.80	mg

^a^Data from USDA Nutrient Data Laboratory

^b, c^Data from Phenol-Explorer 3.0 database

Grapes are the fourth most produced fruit worldwide. The first place is for bananas with 115.75 million tonnes, followed by watermelons with 103.97 million tonnes and apples with 86.14 million tonnes [14]. The world production of grapes was 77.8 million tonnes in 2018, 57% of wine grape, 36% of table grape and 7% of dried grape [15]. However, considering that fresh grapes might not be available everywhere during the whole year, natural supplements obtained from grapes, such as grape beverages or grape extracts may be an interesting alternative to fresh fruit.

Fruit polyphenols have shown antioxidant potential beneficial for the reduction of the effects of oxidative damage during intense exercise in athletes of different disciplines [16, 17]. Polyphenols are poorly absorbed in the human small intestine and undergo extensive biotransformation after ingestion [18, 19]. Evidence supports that biological activities of many polyphenols are actually improved after their biotransformation [20–22]. This process takes time, hence, a prolonged period of polyphenol intake is recommended prior to exercise stress interventions to allow body tissues to adapt to a higher phenolic flux level. That is the reason besides using appropriate outcome measures, long periods are needed to capture such bioactivities [23]. In this context, targeted metabolomics is a suited tool that allows to investigate the shifts of gut-derived metabolites after polyphenol supplementation. Several human trials are revealing an increasing number of metabolites that appear at high concentration levels in the colon and systemic circulation which could be directly associated with polyphenols positive effect against OS [23, 24]. In fact, a systematic review suggested the key role of gut microbiota in controlling the OS during intense exercise [25].

Currently, few papers are available and research designs vary widely regarding to grape polyphenolic supplementation form (drinkable or edible), dosage (acute to multiple weeks and months), type of exercise stress (acute or chronic), profile of subject (trained or untrained), and oxidative stress outcome measures. The aim of this review is to examine the potential effect of these dietary supplements on oxidative stress promoted by exercise in athletes/trained subjects. For this purpose, an evaluation of the available scientific literature has been carried out since it is an important step to determine the efficacy of these polyphenolic based products on the redox status of the athletes. A “dietary supplement” has been considered as a product that is intended to supplement the diet and contains a “dietary ingredient” [26]. In this work, the ingredient refers to the polyphenols present in the grape-based products studied.

### Exercise-induced oxidative stress

Oxidative stress is defined as a result of an imbalance between reactive species production and intrinsic antioxidant defense [27]. For example, athletes participating in one bout of prolonged and intensive exercise such as marathon and ultramarathon race event show acute physiological stress reflected by muscle microtrauma, oxidative stress, inflammation, and gastrointestinal dysfunction [11, 23, 24, 28–34].

The discovery that muscular exercise increases oxidant damage did not occur until the late 1970s [35–37]. Although the biological significance of this finding was unclear, these pioneering studies generated interest for future investigations to examine the important role that radicals, reactive nitrogen species (RNS), and reactive oxygen species (ROS) play in skeletal muscle and other metabolically active organs during exercise. Indeed, growing evidence reveals that while uncontrolled production of RNS and ROS can damage cells, intracellular oxidants also play important regulatory roles in the modulation of skeletal muscle force production, regulation of cell signaling pathways, and control of gene expression [35, 38–42].

Although a multitude of free radicals exists, those derived from either oxygen and/or nitrogen represent the most important class of radicals generated in living systems [43, 44]. The radicals themselves as well as the nonradical species created via interaction with free radicals are collectively referred to as reactive oxygen/nitrogen species (RONS) [45].

The redox activity of RONS plays a critical role in cell signaling and exercise adaptation. It is a phenomenon widely known as hormesis, which means that low levels of stress promote adaptation and therefore, protection from subsequent stress [46, 47]. Exercise-induced RONS act as signaling molecules for the beneficial effects in response to exercise training. RONS produced during muscle contractions are responsible for key adaptations to exercise training as mitochondrial biogenesis [48], endogenous antioxidant enzyme upregulation [49], muscle hypertrophy [50] and glucose uptake by the skeletal muscle [51].

However, at very high concentrations, free radicals instead of being advantageous they can have detrimental effects [46]. During heavy endurance training, endogenous antioxidant capacity cannot counteract the increasingly high RONS generation, resulting in a state of OS and subsequent cellular damage [52].

OS can be basically estimated measuring free radicals, radical mediated damages on lipids, proteins or deoxyribonucleic acid (DNA) molecules and performing the total antioxidant capacity.

The results of free radicals must be interpreted with caution because of the short life of the ROS, their strong ability to react and their low concentration.

Regarding lipid peroxidation, the conventional oxidative stress marker is malondialdehyde (MDA) which is produced during fatty acid oxidation. This product is measured by its reaction with thiobarbituric acid which generates thiobarbituric acid reactive substances (TBARS) in blood samples. F2-isoprostanes are also analyzed to estimate the damage on lipids. They are produced by non-cyclooxygenase dependent peroxidation of arachidonic acid. They are stable products released into circulation before the hydrolyzed form is excreted in urine. Different methodologies can be used for their analysis such as Gas chromatography-Mass Spectrometry (GC–MS), High Performance Liquid Chromatography/Gas Chromatography-Mass Spectrometry (HPLC/GC–MS), Gas Chromatography-tandem Mass Spectrometry (GC-tandem MS), and more recently some immunoassay techniques [53].

Free radical induced modification of proteins causes the formation of carbonyl groups into amino acid side chains. An increase of carbonyls is linked to oxidative stress in blood samples.

For DNA modification quantification, the most used marker is the nucleotide 8-hydroxy-2′-deoxyguanosine (8-OHdG), excreted via blood and urine which is produced by free radicals-induced guanine oxidation and analyzed by Enzyme-Linked ImmunoSorbent Assay (ELISA assays), High Performance Liquid Chromatography-Electrochemical Detection HPLC-ECD or HPLC/GC-MS methods [53].

Regarding total antioxidant capacity, is commonly assessed via the application of one of several antioxidant capacity assays: trolox equivalent antioxidant capacity (TEAC assay), ferric reducing ability of plasma (FRAP assay), 2,2-diphenyl-1-picrylhydrazyl (DPPH assay) and oxygen radical absorbance capacity (ORAC) and/or measurement of changes in specific antioxidant enzymatic activity like superoxide dismutase (SOD), catalase (CAT) and glutathione peroxidase (GPX) by enzymatic assays.

The use of antioxidant supplements for ameliorating the exercise-induced RONS has become a current topic as there is considerable evidence that these supplements might not only prevent the toxic effects of RONS, but also blunt their signaling properties responsible for the adaptive responses [54]. While chronic daily use of antioxidant supplements should be avoided, strategic use of these products in and around periods of heavy training/game scheduling is the best approach [55]. Anyway, further research to observe effects of nutritional antioxidant supplements on exercise-induced oxidative stress must be performed [56].

### Polyphenols: a natural source of antioxidants

An antioxidant can be defined as a substance that helps to reduce the severity of OS either by forming a less active radical or by quenching the damaging free radicals chain reaction on substrates such as proteins, lipids, carbohydrates or DNA [57]. Some antioxidants can interact with other antioxidants regenerating their original properties; this mechanism is usually referred to as the “antioxidant network”.

The antioxidants can be endogenous or obtained exogenously as a part of a diet or as a dietary supplement. Some dietary compounds that do not neutralize free radicals but enhance endogenous antioxidant activity may also be classified as antioxidants. While exogenous antioxidant may attenuate intracellular adaptation in response to exercise training, there is no literature to suggest that increasing endogenous antioxidants has this effect [46].

Endogenous antioxidants keep optimal cellular functions and thus systemic health and well-being. However, under some conditions endogenous antioxidants may not be enough, and extra antioxidants may be required to maintain optimal cellular functions. Such a deficit is evident in some individuals during the overloaded period of training or in circumstances where athletes have little time for recovery like in tournament situations. However, available data still do not allow to define the optimal antioxidant intake that would protect overloaded or, even more so, overtrained individuals [58].

Humans have developed highly complex antioxidant systems (enzymatic and non-enzymatic) which work synergistically and together with each other to protect the cells and organ systems of the body against free radical damage.

**Fig. 1:**
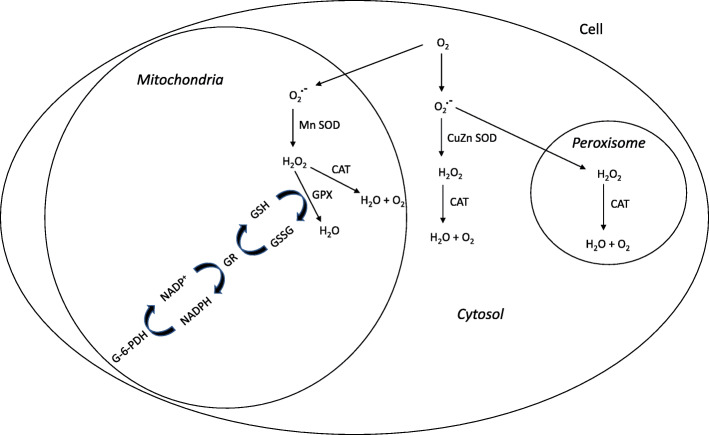
The antioxidant enzyme system in the cell

The most efficient enzymatic antioxidants are superoxide dismutase (SOD), catalase (CAT) and glutathione peroxidase (GPX). In Fig. 1, the antioxidant enzyme system in the cell is shown.

SOD is the major defense upon superoxide radicals and is the first barrier protection against oxidative stress in the cell. SOD represents a group of enzymes that catalyse the dismutation of O_2_^**.-**^ and the formation of hydrogen peroxide (H_2_O_2_). Manganese (Mn) is a cofactor of Mn-SOD form, present in the mitochondria and copper (Cu) and zinc (Zn), are cofactors present in cytosol [57]. In muscular cells, 65–85% of SOD activity is done in the cytosol [59]. Furthermore, CAT is responsible of the decomposition of H_2_O_2_ to form water (H_2_O) and oxygen (O_2_) in the cell. This antioxidative enzyme is widely distributed in the cell, with the majority of the activity occurring in the mitochondria and peroxisomes [59]. With high ROS concentration and an increase in oxygen consumption during exercise, the enzyme GPX, present in cell cytosol and mitochondria, is activated to remove hydrogen peroxide from the cell [60]. The reaction uses reduced glutathione (GSH) and transforms it into oxidized glutathione (GSSG). GPX and CAT have the same action upon H_2_O_2_, but GPX is more efficient with high ROS concentration and CAT with lower H_2_O_2_ concentration [61, 62]. In response to increased RONS production the antioxidant defense system may be reduced temporarily, but may increase during the recovery period [63, 64] although conflicting findings have been reported [65]. GPX requires several secondary enzymes glutathione reductase (GR) and glucose-6-phosphate dehydrogenase (G-6-PDH) and cofactors GSH and the reduced nicotinamide adenine dinucleotide phosphate (NADPH) to remove H_2_O_2_ from the cell.

By contrast, non-enzymatic antioxidants include vitamin A (retinol) [57], vitamin E (tocopherol) [66], vitamin C (ascorbic acid), thiol antioxidants (glutathione, thioredoxin and lipoic acid), melatonin, carotenoids, micronutrients (iron, copper, zinc, selenium, manganese) which act as enzymatic cofactors and flavonoids, a specific group of polyphenols [67].

**Fig. 2:**
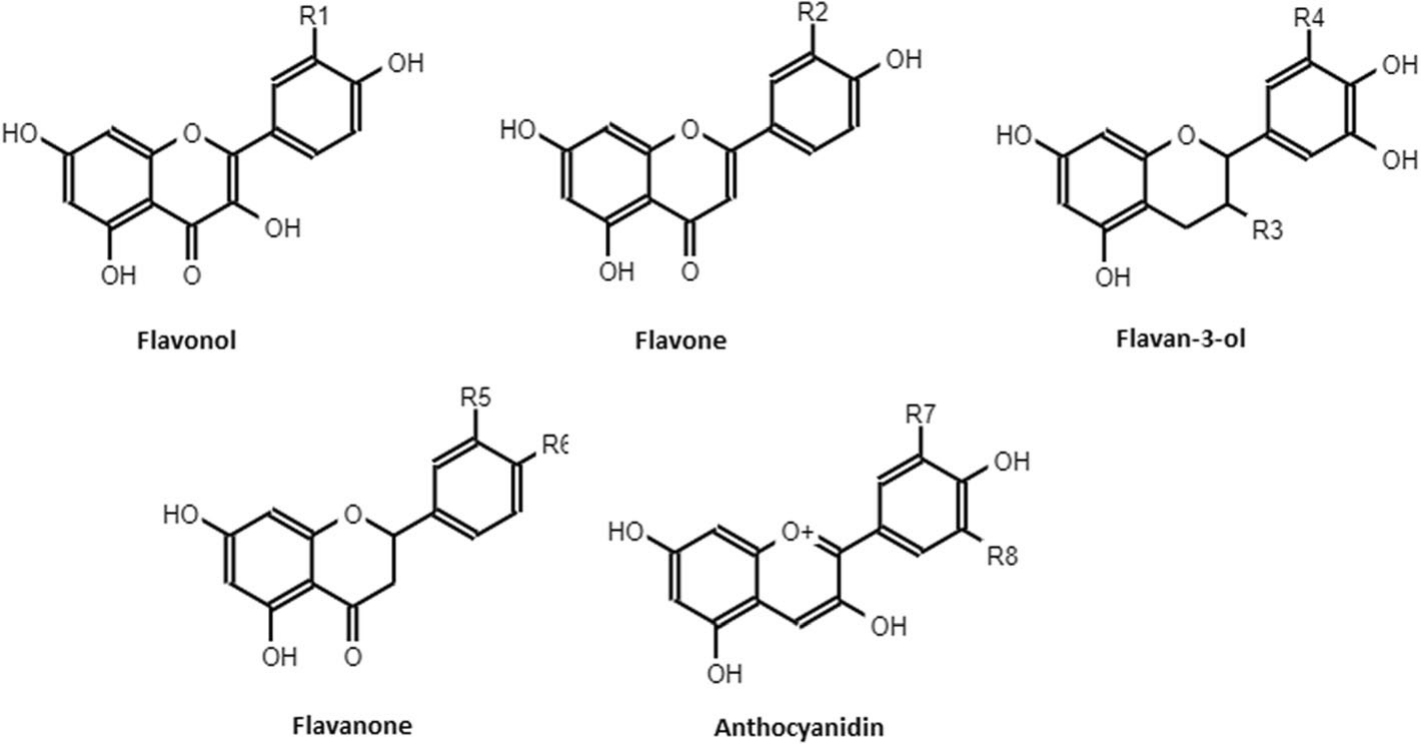
Flavonoid structures. R1 = OH: Quercetin; R1 = H: Kaempferol; R2 = OH: Luteolin; R3 = OH, R4 = H: Catechin; R3 = gallate, R4 = OH: Epigallocatechin-3-gallate; R5 = OH, R6 = OH: Eriodictyol; R5 = H, R6 = OH: Naringenin; R7 = OH, R8 = H: Cyanidin, R7 = OCH_3_, R8 = OCH_3_: Malvidin

Among non-enzymatic antioxidants, polyphenols are a group of phytochemicals that have received great attention of researchers in the last years considering their beneficial effects in the prevention of many chronic diseases [68, 69]. They constitute one of the most numerous and widely distributed groups of natural products in the plant kingdom. Polyphenols can be classified by their origin, biological function, and chemical structure. More than 8000 phenolic structures are currently known, and among them over 4000 flavonoids have been identified [70–72]. The major groups of flavonoids of nutritional interest are the flavonols, the flavones, the flavanols, the flavanones, the anthocyanidins and the isoflavones [73]. See Fig. 2.

Polyphenols have showed to act as a defense against OS caused by excess reactive oxygen species (ROS) [74]. Their potential health benefits as antioxidants is mediated by their functional hydroxyl groups (OH) that determine the ROS synthesis suppression, the chelation of trace elements responsible for free radical generation, the scavenging ROS and the improvement of antioxidant defenses [75, 76].

Commonly, grapes and grape based products are recognized as natural food products with strong antioxidant activity precisely due to their high content in polyphenolic compounds [77].

In fact, some nutraceuticals based on polyphenols have already showed efficacy in reducing the oxidized low-density lipoprotein levels and trimethylamine N-oxide (TMAO is a recognized biomarker of increased cardiovascular risk) serum levels in overweight/obese adults [78] and the gut microbiota remodeling [79]. At the same time, these products have also demonstrated a reduced OS and the oxidative damage at muscular level and improved the muscle performance but in aged rats [80].

Table 2 provides a summary of the different polyphenol families found in grapes.

**Table 2:** Classification of major polyphenols present in grapes and derivatives

Group	Subclass	Compound
Flavonoids	Flavonols	Quercetin, Kaempferol, Myricetin, Isorhamnetin, Laricitrin, Syringetin
	Flavones	Luteolin, Genistein, Apigenin
	Flavanols	Catechin, Epicatechin, Gallocatechin, Epigallocatechin, Epicatechin gallate, Epigallocatechin gallate
	Flavanones	Eriodictyol, Naringenin, Hesperetin
	Anthocyanins	Cyanidin, Peonidin, Delphinidin, Pelargonidin, Petunidin, Malvidin
	Flavanonols	Taxifolin, Astilbin, Dihydromyricetin-3-0-rhamnoside
	Flavanes	
	Chalcones and Dihydrochalcones	
Phenolic acids	Hydroxybenzoic acids	Parahydroxybenzoic, Protocatechuic, Vanillic, Gallic, Syringic
	Hydroxycinnamic acids	p-Coumaric, Caffeic, Ferulic, Sinapic, Caftaric, p-Coutaric, Fertaric
Tannins	Hydrolyzable tannins	Gallotannins, Ellagitannins
	Condensed tannins	Proanthocyanidins
Stilbenes		Resveratrol, Viniferins, Piceid, Piceatanol, Astringin, Pterostilbene, Pallidol, Parthenocissin, Ameurensin G
Coumarins		Umbelliferone, Esculetin, Scopoletin
Phenylethanol derivatives		Tyrosol, Hydroxytyrosol
Lignans and neolignans		Isolariciresinol, Secoisolariciresinol, Lariciresinol, Cedrusin

Considering their polyphenolic composition, it is plausible to hypothesize that the strategic supplementation with grape based products may have a positive antioxidant effect in athletes in particular situations. However, pilot studies on the antioxidant capacity of grapes and grape based products with athletes are scarce. Few studies are focused on the consumption of antioxidant supplements obtained from grape based products to reduce the immediate increase of oxidative stress biomarkers.

Table 3 shows a descriptive summary of 12 studies published since 2005 that investigate the effect of supplementation with grape based products on exercise-induced oxidative stress markers and the antioxidant enzymatic system efficiency. The studies collected in Table 3 fulfill the following inclusion criteria: (i) pilot studies conducted with healthy human participants (active or trained subjects), (ii) original studies with an acute or long-term grape supplementation intervention on physiological responses associated with OS produced by exercise, (iii) published until June 2020. Exclusion criteria are animal studies and studies in which no exercise is performed. Wine may be a good option as a product obtained from grapes with an important source of phenolic compounds. However, considering that wine contains alcohol may not be an option for all consumers due to certain disease conditions, religious restrictions, or age, it has not been considered.

**Table 3:** Effects of grape supplementation on exercised-induced oxidative stress

Study	Supplement form	Polyphenolic content	Dosage	Participant characteristics	Exercise protocol	Sampling	Results
Morillas-Ruiz et al., 2005 [81]	Beverage made of black grape (81 g/l), raspberry (93 g/l) and red currant (39 g/l)	Total phenolic content 1.41 mg/l (anthocyanins: 60%, hydroxycinnamic acid esters: 19%, ellagic acid: 13%, flavonols: 6% and stilbenes: 1%)	30 ml/kg (15 min pre-exercise) and 30 ml/kg every 15 min during a 90 min constant-load test	Moderate trained cyclists (*n* = 30)	Bicycle ergometer 90-min exercise at 70% VO2max	Blood samples 30 min pre-exercise and 20 min post-exercise. Urine for 24-h.	↔ TBARS in either the placebo group or the test group; decreased ↓ carbonyls in the group receiving antioxidants; ↑ 8-OHdG in the placebo group
Goncalves, Bezerra, Cristina, Eleutherio, & Bouskela, 2011 [82]	Organic grape juice	5.32 mg/ml polyphenols	300 ml/day for 20 days	Trained male triathletes (n = 10)	30 km cycling, 7 km running, 2 km swimming per day for 20 days	Blood samples before and after 20 days. Fasting 12 h and after 20 h exercise	↑ peak levels of serum insulin; ↑ plasma uric acid; ↑ functional capillary density; ↑ red blood cell velocity;↓ plasma glucose level; ↓SOD; ↓ time required to reach red blood cell velocity during postocclusive reactive hyperemia
Silvestre, Juzwiak, Gollücke, Dourado, & D’Almeida, 2014 [83]	Grape concentrate drink	Total phenolic content 45.8 g GAE / kg; 27.03 g Vitamin C / kg	300 ml (two doses) at breakfast and immediately after exercise	Trained triathletes (n = 6)	100 km of cycling, 6 km of running and 1.5 km of swimming	Fastin blood sample before, immediately after and 1 h after exercise. Overnight fasting	↑ SOD with both test drink and placebo; ↑ GSH greater after 1 h with placebo; ↑ TBARS higher with placebo; ↓ CAT with placebo after 1 h
Toscano et al. 2015 [84]	Purple grape juice	1.82 g/l total phenolic compunds (52.58 mg/l total monomeric anthocyanins)	10 mL/kg/day in two doses/day for 28 days	Recreationally active amateur runners (n = 28)	A time-to-exhaustion exercise test, anaerobic threshold test, and aerobic capacity test were performed, together with assessments of markers of oxidative stress, inflammation, immune response, and muscle injury, performed at baseline and 48 h after the supplementation protocol	Blood before, after 14 days and after 28 days. Fasting 12 h and after 48 h without training	↑ running time-to-exhaustion; no significant improvements in either anaerobic threshold or aerobic capacity; ↑ TAC; ↓ AGP; no effect in the immune response nor in the activity of CK and LDH
Tavares-Toscano et al., 2017 [85]	Purple grape juice	Total phenolic content (mg GAE/l) 1821 ± 101	10 ml/kg/day in two doses/day for 28 days	Recreationally active street runners (*n* = 48 males and *n* = 5 females)	Intense and continuous physical exercise	Blood before and after 28 days. Both sampling: fasting 12 h and after 48 h without training	↑ TAC, ↔ glycaemic profile, ↓ LDL-cholesterol level, ↑ HDL-cholesterol level, ↓ systolic blood pressure, ↔ diastolic and mean blood pressures
de Lima Tavares-Toscano et al., 2019 [87]	Purple grape juice	Total phenolics (mg/l) 3106.6; Flavanols (mg/l) 13.0; Flavonols (mg/l) 5.3; Phenolic acids (mg/l) 83.8; Stilbenes (mg/l) 2.1	A single dose of 10 ml/kg/day	Recreational male runners (*n* = 14)	Running test at 80% VO2max until exhaustion	Blood before (2 h after supplementation) and after exercise	↑ TAC; ↔ MDA, AGP, hs-CRP, CK, LDH
Sadowska-Krępa, Barbara Kłapcińska, & Kimsa, 2008 [88]	Red grape skin extract	The capsule contains 188 mg/g of polyphenols (catechin, gallic acid, quercetin, trans-resveratrol, cis-resveratrol); 35 mg/g of anthocyanidins (malvidin, peonidin, petunidin, delphinidin, cyanidin).	3 capsules of 390 mg /day for 6 weeks	Physical education students (n = 14)	Interval-type swimming test (free-style with moderate to high intensity)	Blood sample before, after and 1 h after exercise.	↓ CK; ↔ antioxidant enzyme (SOD, CAT, GSH-Px, GR) activities; ↑ concentrations of non-enzymatic antioxidants (GSH, UA) and TA status; ↑ swim speed at lower heart rate
Lafay et al., 2009 [91]	Grape extract	Total polyphenols (> 90%), total flavanols (> 50%), flavanols monomers and gallic acid (> 12%)	400 mg /day in two capsules at breakfast over 1 month	Elite sportsmen (handball *n* = 10, basketball n = 5, sprint n = 4, and volleyball n = 1).	Effort tests were conducted using the Optojump® system, which allows determining the total physical performance, explosive power, and fatigue.	Blood and urine samples before and after 30 days (fasting > 10 h)	↑ ORAC at day 30; ↓ FRAP value in the placebo group but not in the test group; ↑ urinary isoprostane values in the placebo group but not in the test group; ↓CK ↑ hemoglobin levels in the test group; no differences in the effort test; differences in physical performance among sport disciplines
Skarpańska-Stejnborn, Basta, Pilaczyńska-Szcześniak, & Horoszkiewicz-Hassan, 2010 [89]	Grape derived product containing black wine grape peel and seed extract	One capsule contains 188 mg/g of polyphenols (catechin, gallic acid, quercetin, trans-resveratrol, cis-resveratrol) and 35 mg/g of anthocyans (malvidin, peomidin, petunidin, delphinidin, cyanidin)	One gelatin capsule/day of 367 mg for 6 weeks	Trained male rowers (*n* = 22)	Physical exercise test on the rowing ergometer; varying between 40 and 90% of maximal aerobic power	Blood sample before, 1 min after the test completion and after 24 h	↑ TAC; insignificant increase in SOD; ↓ GPX; ↓lipid peroxidation product levels
Taghizadeh, Malekian, Memarzadeh, Mohammadi, & Asemi, 2016 [90]	Grape seed extract	No info	300 mg twice a day for 8 weeks	Female volleyball players (*n* = 40)	No specific test	Blood before and after. Fasting 12 h	↑ GSH; ↓ MDA; ↓ insulin; ↓ homeostasis model of assessment for insulin resistance (HOMA-IR); ↑ insulin sensitivity check index (QUICKI); no significant effects on CK; TAC; NO; FPG and lipid concentrations
Cases et al., 2017 [92]	An innovative polyphenol-based food supplement	Bioactives 410 mg/1000 mg; Polyphenolic bioactives 290 mg/1000 mg; Caffeine 120 mg/100 mg	2 capsules of 500 mg, 60 min before the exercise protocol	Recreationally active male athletes (*n* = 15)	The Wingate test	Blood before, after and at recovery period	↑ oxidative homeostasis; ↑SOD; ↑GSH; ↑CAT; ↑ total power output; ↑ maximal peak power output; ↑ average power developed; ↔ fatigue; ↔ heart rate; ↑ oxidative homeostasis
D’unienville et al., 2019 [93]	Dried grapes with almonds and dried cranberries	One product mix contains 560.3 mg of total polyphenols; 14.84 mg of flavonoids	One product mix/day (2550 KJ/day of energy) for 4 weeks (75 g of raw, natural, unsalted almonds + 25 g of dried grapes (sultanas) + 25 g of dried cranberries)	Trained male cyclists/triathletes (*n* = 96)	Endurance exercise performance	Blood and urine samples	Results to be published

*VO2max* maximal oxygen uptake, *TBARS* Thiobarbituric acid reactive substances, *8-OHdG* 8-hydroxy deoxy guanosine, *SOD* Superoxide dismutase, *GSH* Glutathione, *CAT* Catalase, *TAC* Total antioxidant capacity, *AGP* α1-acid glycoprotein, *CK* Creatine kinase, *LDH* Lactate dehydrogenase, *LDL* Low-density lipoproteins, *HDL* High-density lipoproteins, *GR* Glutathione reductase, *UA* Uric acid, *TA* Total antioxidant, *ORAC* Oxygen radical absorbance capacity, *FRAP* Ferric reducing ability of plasma, *MDA* Malondialdehyde, *NO* Nitric oxide, *FPG* Fasting plasma glucose

### Grape polyphenols supplementation effect

Among the studies found, six of the products are beverages made with grape and the rest are grape extracts and only one is referred to dried grapes.

#### Grape beverage supplements

Within the beverages, one is a grape beverage but mixed with raspberry and red currant [81], another one a grape beverage specified as organic [82], two of them are grape concentrate drinks [83, 84] and the last two a purple grape juice [85].

Regarding the polyphenolic content, the studies show a wide number of dosages. Morillas-Ruiz et al.dose range. [81] established an acute dose of the beverage at 30 ml/kg before doing exercise and 30 ml/kg every 15 min during 90 min of constant-load test on a bicycle ergometer. Considering the total phenolic content of 1.41 mg/l of the beverage, this means a total polyphenolic dose of 17.76 mg for a moderate trained cyclist with 70 kg for example. In this study no significant difference from basal to post-exercise period was detected for plasma thiobarbituric acid reactive substances analysis (TBARS) neither in the placebo group (*n* = 13) nor the group receiving the antioxidant supplemented beverage (n = 13). This could be explained by a not high enough intensity exercise to alter the redox state or by the adaptation on antioxidant defenses in well-trained subjects. However, the antioxidant supplementation had a beneficial effect on the oxidation of proteins induced by exercise and reduced this index. In fact, the group receiving antioxidants obtained a 29% reduction in protein carbonyls. However, an unexpected result was obtained for 8-oxo-7,8-dihydro-2′ deoxyguanosine (8-OHdG) in urine with a greater decrease in comparison to the study group. Despite these results, the authors defend the usefulness of 8-OHdG determination as a sensitive index of the relationship between exercise and oxidative stress and demonstrate that antioxidant-supplemented beverages reduce 8-OHdG excretion following a 90 min exercise protocol.

Other authors established an intake of 300 ml/day of an organic grape juice for 20 days [82]. Considering the total phenolic content of 5.32 mg/ml, the total ingestion of polyphenols per day was 1.59 g for each trained male triathlete (*n* = 10). In this case, the results showed a decreased superoxidase dismutase (SOD) activity in erythrocytes activity after 20 days. SOD is a cytosolic antioxidant enzyme responsible for superoxide anion radical dismutation into oxygen and hydrogen peroxide and is sensitive to the intake of polyphenols in humans. The reduction before (baseline) and after 20 days was 27.8 ± 6.3 to 24.3 ± 2.5 U/mg protein. The authors attributed this decrease to the reduction of intra- and extracellular oxidative imbalances.

The effect of the same volume of 300 ml/day of a grape concentrate juice (*Vitis labrusca*) with a total phenolic content of 45.8 g GAE (Gallic Acid Equivalents)/kg beverage was studied by Silvestre et al. [83]. In this case, the total intake of polyphenols for each trained triathlete (*n* = 6) was 3 g. The acute intake was in two equal doses before and after the training. The results showed a significant increase in SOD in the blood samples regardless of the drink consumed (grape drink or placebo). A lower increase in reduced glutathione (GSH) levels in the test group in comparison to the placebo group was obtained. This result may indicate a lower oxidation of GSH to GSSG, oxidized glutathione, due to the action of glutathione peroxidase (GPX) or even more efficient synthesis by glutathione reductase. Besides, higher values in TBARS value with placebo in comparison to the grape concentrate drink were obtained just after the exercise and after one hour. This means a lower value in this oxidative stress marker related to lipid peroxidation when grape concentrate drink is consumed. But the antioxidant enzyme catalase (CAT) activity remained stable in the group that consumed the beverage. The authors suggest that the studies on the CAT response to exercise have shown conflicting results especially to a single exercise session. The study concludes that TBARS, CAT and GSH values suggest that this grape concentrate drink presents potential to modulate exercise-induced oxidative stress.

In another study Tavares-Toscano et al. [85] provided purple grape juice to recreationally active street runners (*n* = 53) at a total dose of 10 ml/kg/day for 28 days. Considering the total phenolic content of 1821 ± 101 mg GAE/l the total intake of polyphenols reached the total of 1.27 g per day and 35.69 g polyphenols after 28 days. The results showed a significant increase, up to 39% in the plasma antioxidant activity after 28 days. In this case the total antioxidant capacity (TAC) was evaluated in the plasma by evaluating the radical scavenging according to the α, α-diphenyl-β-picrylhydrazyl (DPPH) method. This analytical method is used to determine the TAC of a compound, an extract or other biological sources by using a stable free radical DPPH. The assay is based on the measurement of the scavenging capacity of antioxidants towards it [86]. The authors showed a deep characterization of the grape juice. They did not analyze any oxidative stress markers, but showed an increase in high density lipoprotein-cholesterol (HDL-cholesterol) fraction and a decreased low-density lipoprotein-cholesterol (LDL-cholesterol) fraction demonstrating that grape juice may enhance the benefits of physical training,

The same author [84] demonstrated, with the same beverage and dosage in recreationally active amateur runners (*n* = 28), an increase in TAC by 38% in comparison to the control group after 28 days. Besides the malondialdehyde (MDA) data indicated that grape juice supplementation did not prevent lipid peroxidation in athletes, but the increase was lower than in the group with no grape juice.

Tavares-Tocano et al. [87] also showed that a single dose of purple grape juice of 10 ml/kg with a concentration of 3106.6 mg/l was able to promote increased plasma antioxidant activity in recreational male runners, but did not change the plasma concentration of lipid peroxidation by MDA.

#### Grape extract supplements

Studies found with this type of supplements are focused on an extract obtained from the grape’ skin [88], extracts obtained from grape seeds [89, 90], the whole grape [91], an innovative polyphenol-based food supplement based on a grape extract [92] and dried grapes with almonds and dried cranberries [93].

Concerning the edible grape products, to the best of our knowledge the first study that analyzed the effect of grape polyphenols supplementation on the blood antioxidant status was in 2008 [88]. In this study an intake of 3 capsules containing 390 mg of red grape skin extract per day for 6 weeks to fourteen physical education students (*n* = 14) was established. This dosage means 0.22 g polyphenols per day and a total 9.24 g after the 6 weeks. The results showed an insignificant modification of antioxidant enzyme: SOD, CAT, GSH and glutathione reductase (GR) activities, concentrations of non-enzymatic antioxidants: GSH and uric acid (UA) and total antioxidant status (TAS). However, the authors indicated that the supplementation with the alcohol-free red wine grape polyphenolic extract might influence the attenuation of the post-exercise release creatine kinase (CK) into the blood.

Lafay et al. [91] established a dosage of 400 mg of a commercial grape extract over a month period for elite sportsmen (*n* = 20). In this case, no information regarding the total polyphenolic content was given. The authors showed that consumption of grape extract standardized in flavanols permits to ameliorate the oxidative stress/antioxidant status balance in elite athletes during a competition period, and to enhance physical performance in one category of sportsmen (handball). Besides the administration of grape extract decreased the plasma CK concentration and increased the hemoglobin (Hb) level in plasma suggesting a protection of cells against oxidative stress damage.

In another work [86] the authors gave to each male rower (*n* = 22) one gelatin capsule containing a commercial black grape extract three times a day for six weeks what results an amount of 0.21 g polyphenols per day and a total of 8.69 g total polyphenols after the 43 days. The study revealed that this preparation and doses contributed to a significant increase in plasma TAC and to an insignificant increase in SOD, as well as a lower GSH activity and reduce concentration in TBARS.

Taghizadeh et al. in a pilot study [90] tested the effect of a grape seed extract (GSE) on female volleyball players (*n* = 40). The dosage was 300 mg of GSE twice a day for 8 weeks. No information about the polyphenol content was given but the results showed a significant rise in plasma GSH and a significant decrease in MDA. Besides, the players who received GSE exhibited a significant decrease serum insulin concentration. On the other hand, the administration of GSE had no significant effects on parameters like creatine kinase (CK) or TAC when compared with the administration of the placebo.

Another pilot study [92] is developed with an acute intake of 1000 mg of a commercial grape supplement with pomegranate in two 500 mg capsules 60 min before the start of an intense and continuous cycling exercise (Wingate test). The polyphenol content was 29 g/100 g which results a dose of 0.29 g polyphenols. The study resulted in an increase in SOD, GSH and CAT activity, which remained stable until the end of the recovery period. The authors explained that in comparison with the placebo group the subjects supplemented showed no need to mobilize more antioxidant defenses before the exercise because and that the supplement probably contributed to spare oxidative homeostasis.

Finally, it must be pointed out the protocol [93] established for a pilot study that includes a product mix made of dried grapes with almonds and dried cranberries. No results are given but the authors describe the necessity of studying the F2-isoprostanes as a lipid peroxidation biomarker for oxidative stress.

## Conclusions

Supplementation with grape polyphenols seems to have a positive effect against oxidative stress. These effects are dependent on the supplement dose, the length of the supplementation period or the polyphenolic profile (total polyphenol content and the distribution among polyphenolic families). Besides, according to several reports, it appears that the type and intensity of exercise can affect the response of the blood antioxidant defense system, just as the training status of the athlete, or the sport discipline. Considering the supplementation dosage in these studies it seems unlikely athletes would gain enough quantity of polyphenols from diet. Therefore, grape-based polyphenol concentrated products would be an interesting approach.

Moreover, inter-individual variability the age, sex, diet, environment factors, exercise protocols and even variability in gene expression could influence the polyphenols bioavailability and physiological responses to oxidative stress. Given the promising evidence, although still limited, more pilot studies on effect of grape polyphenols on the oxidative stress produced by sport should be conducted to determine the optimal concentration, dosage and effect on the oxidative stress for target athletes.

## Data Availability

All data analyzed in this review are included in the cited articles.

## Abbreviations

NCD: Noncommunicable diseases
OS: Oxidative stress
RNS: Reactive nitrogen species
ROS: Reactive oxygen species
RONS: Reactive oxygen/nitrogen species
DNA: Deoxyribonucleic acid
MDA: Malodialdehyde
TBARS: Thiobarbituric acid reactive substances
GC–MS: Gas Chromatography-Mass Spectrometry
HPLC/GC–MS: High Performance Liquid Chromatography/Gas Chromatography-Mass Spectrometry
GC-tandem MS: Gas Chromatography-tandem Mass Spectrometry
ELISA: Enzyme-Linked ImmunoSorbent Assay
HPLC-ECD: High Performance Liquid Chromatography-Electrochemical Detection
8-OHdG: 8-hydroxy-2′-deoxyguanosine
TEAC: Trolox equivalent antioxidant capacity
FRAP: Ferric reducing ability of plasma
DPPH: 2, 2-diphenyl-1-picrylhydrazyl
ORAC: Oxygen radical absorbance capacity
SOD: Superoxide dismutase
CAT: Catalase
GPX: Glutathione peroxidase
H_2_O_2_: Hydrogen peroxide
H_2_O: Water
O_2_: Oxygen
GR: Glutathione reductase
G-6-PDH: Glucose-6-phosphate dehydrogenase
NADPH: Reduced nicotinamide adenine dinucleotide phosphate
TMAO: Trimethylamine N-oxide
VO2max: Maximal oxygen uptake
GSH: Glutathione
TAC: Total antioxidant capacity
AGP: α1-acid glycoprotein
CK: Creatine kinase
LDH: Lactate dehydrogenase
LDL: Low-density lipoproteins
HDL: High-density lipoproteins
UA: Uric acid
TA: Total antioxidant
NO: Nitric oxide
FPG: Fasting plasma glucose
